# Extracting actigraphy-based walking features with structured functional principal components

**DOI:** 10.1088/1361-6579/ad65b2

**Published:** 2024-08-02

**Authors:** Verena Werkmann, Nancy W Glynn, Jaroslaw Harezlak

**Affiliations:** 1 School of Public Health, Department of Epidemiology and Biostatistics, Indiana University, Bloomington, IN, United States of America; 2 Graduate School of Public Health, Department of Epidemiology, Center for Aging and Population Health, University of Pittsburgh, Pittsburgh, PA, United States of America

**Keywords:** accelerometry data, functional data analysis, functional principal component analysis, multilevel functional data

## Abstract

*Objective.* We extract walking features from raw accelerometry data while accounting for varying cadence and commonality of features among subjects. Walking is the most performed type of physical activity. Thus, we explore if an individual’s physical health is related to these walking features. *Approach.* We use data collected using ActiGraph GT3X+ devices (sampling rate = 80 Hz) as part of the developmental epidemiologic cohort study, *I* = 48, age = $78.7\pm5.7$ years, 45.8% women. We apply structured functional principal component analysis (SFPCA) to extract features from walking signals on both, the subject-specific and the subject-spectrum-specific level of a fast-paced 400 m walk, an indicator of aerobic fitness in older adults. We also use the subject-specific level feature scores to study their associations with age and physical performance measures. Specifically, we transform the raw data into the frequency domain by applying local Fast Fourier Transform to obtain the walking spectra. SFPCA decomposes these spectra into easily interpretable walking features expressed as cadence and acceleration, which can be related to physical performance. *Main results.* We found that five subject-specific and 19 subject-spectrum-specific level features explained more than 85% of their respective level variation, thus significantly reducing the complexity of the data. Our results show that 54% of the total data variation arises at the subject-specific and 46% at the subject-spectrum-specific level. Moreover, we found that higher acceleration magnitude at the cadence was associated with younger age, lower BMI, faster average cadence and higher short physical performance battery scores. Lower acceleration magnitude at the cadence and higher acceleration magnitude at cadence multiples 2.5 and 3.5 are related to older age and higher blood pressure. *Significance.* SFPCA extracted subject-specific level empirical walking features which were meaningfully associated with several health indicators and younger age. Thus, an individual’s walking pattern could shed light on subclinical stages of somatic diseases.

## 1. Introduction

**Figure 1.**
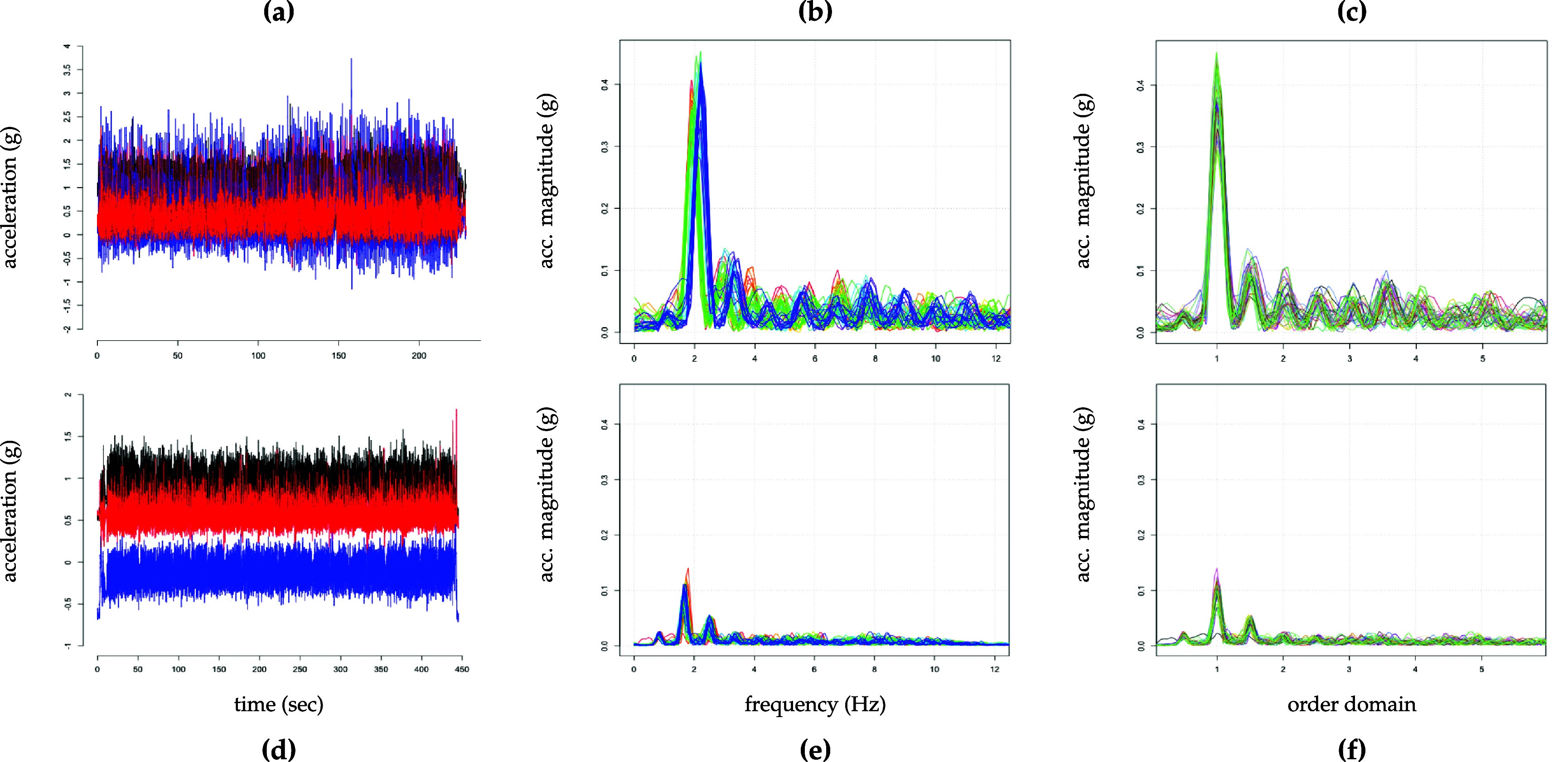
Raw time series and spectra for two participants. Panel (a) raw data, (b) FFT spectra, (c) realigned spectra for Participant A. Panel (d) raw data, (e) FFT spectra, (f) realigned spectra for Participant B.

Walking is the most accessible form of physical activity. There is no need for equipment and everyone can participate at their own pace. As different people walk more or less athletically, walking features are likely predictors of physical health. We might think about this relationship as the more ‘smoothly’ someone walks, the better their physical health. However, before we are able to extract and analyze *walking features*, we first have to obtain *walking data* which is usually done via accelerometers. These wearable devices typically record a signal between 10–100 times per second generating high-frequency time series data. This continuously sampled time record^3^ 3See figure 1, graphs (a) and (d) for raw triaxial signals corresponding to the walking signals collected from two participants. is quite dense which makes it nearly impossible to identify any walking characteristics without further transformations. One such transformation is the *fast Fourier transform* (FFT) (see Cooley and Tukey 1965), essentially splitting the high frequency time series into several-second-intervals in the frequency domain so that individual *functions* are obtained. Thus, preprocessed accelerometry data can be considered functional data where one observation corresponds to a curve which is called spectrum in this specific application^4^ 4See figure 1, graphs (b) and (e) for spectra plots corresponding to two participants’ walks, one curve corresponds to one spectrum.. It is generally expected that these spectra are similar in shape. (Functional) principal component analysis (PCA) makes use of this characteristic to facilitate a reduction in dimension. This decrease in the complexity of the data is achieved by generating linear combinations of the original variables that are uncorrelated but retain as much of the variability in the dataset as possible (see, e.g. Jolliffe and Cadima 2016). In our application, these new variables can then be interpreted as walking features preserving as much information from a walk as possible.

The underlying accelerometry data is part of the Developmental Epidemiologic Cohort Study (DECOS) (see Lange-Maia *et al* 2015), specifically the fast-paced 400 m walk performed by older adults. After preprocessing, the data exhibits a hierarchical structure: for each participant, a particular number of walking spectra in the frequency domain is obtained by applying local FFT to the original high-dimensional time series (spectra *nested within* each participant).

For analyzing such functional data displaying the above described hierarchy, several *multilevel* functional PCA methods have been developed (see e.g. Di *et al* 2009, Greven *et al* 2011) to extract functional principal components (FPC) on the different levels. We apply *Structured Functional PCA* (SFPCA) (see Shou *et al* 2015) to extract features from walking signals on both, the subject- and the subject-spectrum level of the fast-paced 400 m walk. SFPCA allows for multi-way nested and crossed sampling designs, i.e. it is possible to include more than two levels of nesting or crossing to expand the hierarchy. *Structured functional models* thus admit two sampling schemes and two or more random processes which renders SFPCA a generalization of Di *et al* (2009)’s multilevel functional PCA (MFPCA) and Greven *et al* (2011)’s longitudinal functional PCA (LFPCA). MFPCA models the data as a two-way nested model where the data covariance is separated into the between- and within-subject covariance function, and LFPCA takes a similar approach modeling repeated functional observations.

Specifically, the DECOS data can be modeled via a two-way functional nested design. Both, the first level (subject-specific) and the second level (subject-spectrum-specific) functional process are considered latent; only the outcome function, i.e. the walking spectra are observable. SFPCA offers a tool to parsimoniously model the unobservable functional processes. This complexity-reduced representation of the data is achieved by projecting the data into a lower-dimensional space which is spanned by only the most important basis functions on each level. Transforming the data as described above, facilitates eliminating redundancies while retaining shared characteristics on both levels.

Walking is a physical activity that can be performed by a large segment of the population that is quite heterogenous in several aspects pertaining to their overall health. These differences in, e.g. physical fitness, lead to subject-specific walking patterns which are composed of walking features common to all subjects in the sample. How much each of those common features contributes to a subject-specific pattern is specific to an individual. Thus, the walking features themselves as well as their contributions to their subject-specific walking pattern might allow us to learn about individuals’, possibly subclinical, health problems based on their movement.

The remainder of the paper is structured as follows. Section 2 provides the data description and the preprocessing steps performed to prepare the data for the analysis. Section 3 outlines the methods used for the data analysis and section 4 describes the application of these methods to the DECOS data. In section 5, a discussion of the results is offered.

## 2. Data and pre-processing

Eighty-nine community-dwelling older adults (age = $78.7\pm5.7$) were recruited from the Pittsburgh, Pennsylvania area for the National Institute on Aging, Aging Research Evaluating Accelerometry (AREA) project, part of the (DECOS, Lange-Maia *et al* 2015). The University of Pittsburgh Institutional Review Board approved this study and all participants provided written informed consent prior to participation. Participants were excluded if they had a Modified Mini-Mental State Exam (Teng and Chui 1987) score of $ < $80, which was administered at the beginning of the first visit (Lange-Maia *et al* 2015). We use the data of *I* = 48 participants (25 men, 22 women, and one participant with missing value for the variable ‘sex’). All participants were equipped with Actigraph GT3X+ accelerometers placed on the right hip. Devices collected raw accelerometry data along three orthogonal axes with sampling frequency of 80 observations per second (80 Hz). We use the data collected from the fast 400 m walk. Compliance was addressed via visual examination of the data. All participants included in the experiment adhered to the protocol.

The next paragraphs describe the pre-processing steps undertaken to prepare the raw time series data for the analysis (see figure 1, graphs (a) and (d)). With a few exceptions, these steps are in line with those in Fadel *et al* (2020). The first step is to transform the subject-specific raw triaxial signal for each period identified as walking into vector magnitude (VM)

\begin{eqnarray*} vm\left(t\right) = \sqrt{x_1\left(t\right)^2 + x_2\left(t\right)^2 + x_3\left(t\right)^2}\end{eqnarray*}

where $x_1(t)$, $x_2(t)$, and $x_3(t)$ denotes the signal collected along the three axes at time *t*. Next, the spectra for subject $i \in \{1, \dots, I\}$ are computed. This requires separating the VM series into subject-specific several-second non-overlapping windows^5^ 5The narrowest subject-specific window is $\tau_i = 4.5$ s and the widest $\tau_i = 12.5$ s. The window lengths were chosen by exploring different window lengths for the individual-specific walking times. Applying the resulting window lengths, we use 97% of the total number of spectra while at the same time balancing the sample. and converting it from the time to the frequency domain by applying local FFT. We choose subject-specific window lengths to keep the number of resulting spectra per subject $j \in \{1, \dots, J_i\}$ fairly stable across the subjects, $J_i \in \{46,\dots, 50\}$. Following Urbanek *et al* (2018), the local FFT of *vm*(*t*) is

\begin{eqnarray*} X\left(t, f ; \tau_i\right) = \sum_{u = \left[t-\tau_i / 2\right]}^{\left[t+\tau_i / 2\right]} v m\left(u\right) h\left(u\right) \mathrm{e}^{-\mathrm{i} 2 \pi f u / \tau_i}\end{eqnarray*}

with *f* being the frequency index and *τ*_*i*_ denoting a parameter giving the number of observations in the interval centered around *t*. The term *h*(*u*) allows for specification of a window function to reduce potential spectral leakage caused by the windowing. We use the Hann window given by

\begin{eqnarray*} h\left(u ; \tau_i\right) = 0.5\left[1-\cos \left\{2 \pi u /\left(\tau_i-1\right)\right\}\right].\end{eqnarray*}

A spectrum then equals $|X(t, f ; \tau_i)|$. For each several-second window *j* for each subject *i* we thus obtain such an FFT spectrum.

The next step is to determine the subject-spectrum specific dominant or fundamental frequency lying in the range between 1.2 and 4.0 Hz. This range deviates somewhat from the usual assumed frequency of human steps (1.4 Hz–2.5 Hz, see Ji and Pachi 2005) but following Urbanek *et al* (2018) and Fadel *et al* (2020), we choose this more conservative interval to accommodate the slower pace of older adults. For the joint frequency axis across all subjects, we use an equally-spaced grid of 1597 values in the interval between 0 and 39.9 Hz (step size = 0.025 Hz). Figures 1(b) and (e) display the spectra plots for two participants where each line corresponds to one spectrum. Compared to the raw data, the transformation to the frequency domain leads to a much clearer picture. Per subject, the spectra display a roughly similar shape where the fundamental frequency can be visually identified as the location of the maximum of each spectrum curve. The fundamental frequency is also referred to as the *cadence* which is usually reported in steps per second (e.g. 1.2 Hz = 1.2 steps s^−1^). However, plots (b) and (e) in figure 1 also show that there is still a lot of variation in the spectra, both within and between the subjects. To make the spectra more comparable across all subjects, the spectra are aligned at their fundamental frequency and thereby transformed from the frequency to the order domain. This is achieved by scaling the frequency axis per spectrum by the associated fundamental frequency. To put the spectra back on a joint sampling grid in the order domain, linear interpolation is used resulting in an evenly spaced grid between 0 and 11 sampled every 0.01 steps. Lastly, in line with Fadel *et al* (2020), to ensure that signal noise does not distort the subsequent analysis, all spectra are limited to the interval between 0.3 and 5.75 yielding a grid of *p* = 546 sampling points. Also, to facilitate the methods described in the following section, the number of spectra is balanced across all subjects, $J_i = J = 46$. This setup leads to the functional counterpart of hierarchical data where *J* spectrum curves observed in the order domain *t* and sampled on a grid of length *p* are nested within each of the *I* subjects.

## 3. Methods

For modeling the hierarchy in the data, we use a two-way functional nested model which belongs to the class of *structured functional models* introduced by Shou *et al* (2015). Structured functional models are special cases of functional linear mixed models (Guo 2004, Herrick and Morris 2006, Morris and Carroll 2006) and can be categorized according to their sampling scheme (nested or crossed) and the number of levels in the hierarchy. For rendering these intricately structured data accessible, we apply SFPCA (Shou *et al* 2015) to reduce the dimension on both levels and thereby generate new interpretable features. The first-level features can then further be used for statistical modeling.

In a two-way functional nested model, the observed outcome function $Y_{ij}(t)$ for subject $i = 1,\dots,I$ and spectrum $j = 1,\dots,J$ at time $t = 1,\dots,T$ can be decomposed into two latent stochastic processes, a first-level process $X_i(t)$ and a second-level process $U_{ij}(t)$ nested within the first level. Formally, let $X_i(t)$ and $U_{ij}(t)$ be two mutually uncorrelated, zero-mean stochastic processes, then the two-way functional nested model is given by

\begin{eqnarray*} Y_{ij}\left(t\right) = \mu\left(t\right) + X_i\left(t\right) + U_{ij}\left(t\right) + \varepsilon_{ij}\left(t\right), \quad \varepsilon_{ij}\left(t\right) \overset{i.i.d.}{\sim} {WN}\left(0, \sigma^2\right)\end{eqnarray*}

where *Y*_*ij*_ is the functional outcome sampled on the discrete grid with length *p*, $Y_{ij} = \{Y_{ij}(t_1), \dots,$ $ Y_{ij}(t_p)\}^{^{\prime}}_{(1\times p)}$ and $Y = (Y_{11}, \dots, Y_{1J}, \dots, Y_{I1},\dots, Y_{IJ})_{(p\times IJ)}$ and $\mu(t)$ is the overall mean function or fixed effect. Accordingly, $X_i(t)$ and $U_{ij}(t)$ can be considered first- and second-level random effects. Since $X_i(t)$ and $U_{ij}(t)$ are uncorrelated, the observed total variation of $Y_{ij}(t)$, $K_Y(t,s)$, can be split up into the two level-specific covariances as

\begin{eqnarray*} \begin{gathered} \begin{split} K_Y\left(t,s\right) &amp; = K_X\left(t,s\right) + K_U\left(t,s\right) + \sigma^2_\varepsilon \mathcal I_{\left\{s = t\right\}}\\ &amp; = \textrm{Cov}\left[X_i\left(t\right), X_i\left(s\right)\right] + \textrm{Cov}\left[U_{ij}\left(t\right), U_{ij}\left(s\right)\right] + \textrm{Cov}\left[\varepsilon_{ij}\left(t\right), \varepsilon_{ij}\left(s\right)\right]. \end{split} \end{gathered}\end{eqnarray*}

By the Karhunen-Loève theorem, equation (4) can be decomposed as follows

\begin{eqnarray*} Y_{i j}\left(t\right) = \mu\left(t\right) + \sum_{k = 1}^{\infty} \phi_{k}^{X}\left(t\right) \xi_{i k}^{X}+\sum_{\ell = 1}^{\infty} \phi_{\ell}^{U}\left(t\right) \xi_{ij \ell}^{U} + \varepsilon_{ij}\left(t\right),\end{eqnarray*}

where $\phi_{k}^{X}(t)$ and $\phi_{\ell}^{U}(t)$ denote the eigenfunctions of $K_X(t,s)$ and $ K_U(t,s)$, respectively. The mutually independent random variables $\xi_{i k}^{X}$ and $\xi_{ij\ell}^{U}$ are referred to as *functional principal scores* (FP scores) and can be estimated as

\begin{eqnarray*} \widehat\xi\,_{i k}^{X} = \int X_i\left(t\right) \phi_{k}^{X}\left(t\right) \mathrm{d}t,\quad \widehat\xi\,_{i j \ell}^{U} = \int U_{ij}\left(t\right) \phi_{\ell}^{U}\left(t\right) \mathrm{d}t.\end{eqnarray*}

$\widehat\xi_{i k}^{X}$ and $\widehat\xi_{i j \ell}^{U}$ have zero mean and variance equal to the associated eigenvalue $\lambda^X_k$ and $\lambda^U_\ell$, respectively. Generally used in the single-level case (see Müller and Stadtmüller 2005), the expressions in equation (7), however, do not take into account the respective other level or the variance of the (level-specific) noise component when estimating the level-specific FP scores. This approach seems only reasonable when the level data are perfectly separated and observed. In the following section, we outline the conditional expectation (PACE) estimator for the FP scores which can capture both, the impact of the respective other level as well as the variance of the noise component. Moreover, we review the level-specific eigendecomposition of the covariance operators $K_X(t,s)$ and $ K_U(t,s)$ performed for extracting signals and reducing data complexity. The subsequent section discusses the Method of Moments (MoM) approach used for estimating these covariance operators.

### 3.1. Level-specific spectral decomposition

Eigendecomposition is performed on the level-specific covariance functions $K_X(t,s)$ and $ K_U(t,s)$ so that each is decomposed into its respective eigenvalues and eigenfunctions. The first *N_X_* and *N_U_* eigenfunctions of $ K_X(t,s)$ and $ K_U(t,s)$ contain most of the information about the level-specific processes $X_i(t)$ and $U_{ij}(t)$ while the remaining $p-N_X$ and $p-N_U$ eigenfunctions primarily add noise. The Karhunen–Loève decomposition allows for reducing the dimensionality of the data by truncating the sums in equation (6) as follows

\begin{eqnarray*} Y_{i j}\left(t\right) = \mu\left(t\right) + \sum_{k = 1}^{N_X} \phi_{k}^{X}\left(t\right) \xi_{i k}^{X}+\sum_{\ell = 1}^{N_U} \phi_{\ell}^{U}\left(t\right) \xi_{ij \ell}^{U}+ \varepsilon_{ij}\left(t\right).\end{eqnarray*}

In the literature, several procedures have been proposed to select *N_X_* and *N_U_*, for example information criteria such as different versions of a functional data-adapted AIC and BIC (Yao *et al* 2005, Li *et al* 2013) or cross-validation-based methods (Rice and Silverman 1991). Other intuitive and computationally less costly alternatives (see, e.g. Crainiceanu *et al* 2009, Greven *et al* 2011) rely on the cumulative percentage of explained variance (CPV) threshold,

\begin{eqnarray*} \textrm{CPV}\left(N_X\right) = \sum_{k = 1}^{N_X} \lambda^X_k/\sum_{k = 1}^\infty \lambda^X_k, \quad \textrm{CPV}\left(N_U\right) = \sum_{m = 1}^{N_U} \lambda^U_m/\sum_{m = 1}^\infty \lambda^U_m.\end{eqnarray*}

In single-level FPCA, eigenvalue $\lambda^X_k$ serves as the variance explained by eigenfunction $\phi^X_k$. In SFPCA, *K_X_* and *K_U_* are decomposed separately, therefore $\lambda^X_k$ and $\lambda^U_\ell$ have the same interpretation for the respective level. Consequently, *N_X_* (*N_U_*) can be chosen such that $\textrm{CPV}(N_X) \unicode{x2A7E} q$ ($\textrm{CPV}(N_U) \unicode{x2A7E} q$) where *q* is a pre-specified proportion of the variance of $X_i(t)$ ($U_{ij}(t)$). The eigenfunctions $\phi_{k}^{X}$ and $\phi_{\ell}^{U}$ are then referred to as FPCs (see, e.g. Klepsch *et al* 2017).

**Figure 2.**
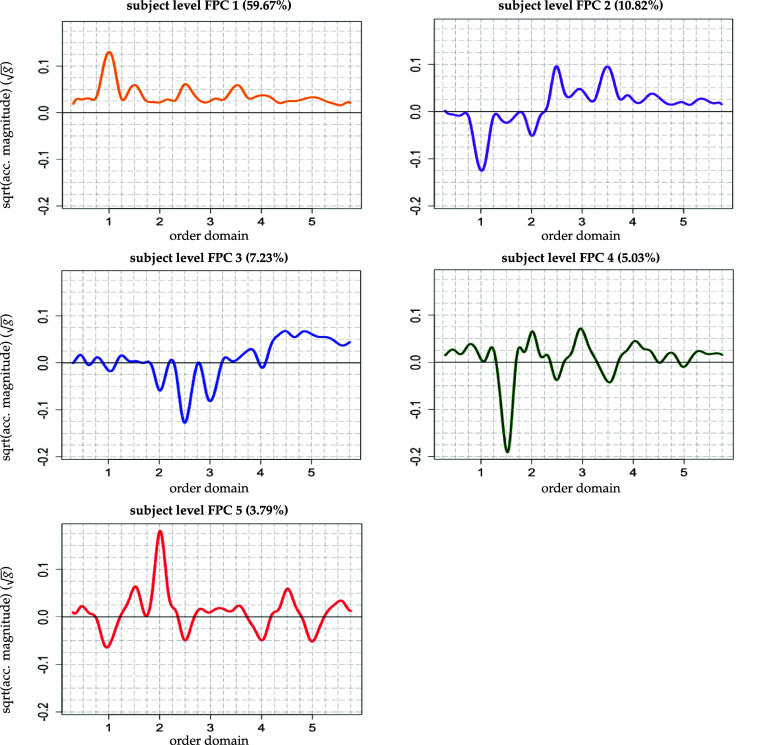
Subject-specific level functional principal components of the square root transformed spectra (corresponding proportion of explained total variance in parentheses). Plots start at 0.3 and end at 5.75 in the order domain.

Empirically, the truncation in equation (8) reduces repetitions in the spectra while retaining shared characteristics of the walking data contained in the FPCs. Thus, the *N_X_* subject-specific FPCs represent *walking features* on the individual level providing a more accessible characterization than *I* subject-specific spectrum plots displaying *J* curves each (compare figure 1 versus figure 2).

The truncated set of level-specific FPCs maps the data to the FP scores $\left\{{{\xi}}_{i}^{X}, {{\xi}}_{ij }^{U}\right\}$ as shown in equation (7). The level-specific FP scores acquire the maximum possible variability from the level-specific data but are of considerably lower dimension because usually $N_X, N_U \ll p$. In a hierarchical framework, the FP scores can be *jointly* obtained as the *best linear unbiased predictors* (BLUP) of $\left\{{{\xi}}_{i}^{X}, {{\xi}}_{ij }^{U}\right\}$ in the following matrix formulation of equation (8)

\begin{eqnarray*} {Y}_{i j} = \widehat{\mu}\left(t\right)+{\widehat{\Phi}_{N_X}}^{X} {\xi}_{i}^{X}+{\widehat{\Phi}_{N_U}}^{U} {\xi}_{i j}^{U}, \quad {\xi}_{i}^{X} \sim \mathcal N\left(0, \widehat{\Lambda}_{N_X}^{X}\right), \;{\xi}_{ij}^{U} \sim \mathcal N \left(0, {\widehat\Lambda}_{N_U}^{U}\right),\end{eqnarray*}

where ${\widehat\Lambda}_{N_X}^{X} = \textrm{diag}(\widehat\lambda^X_1, \dots, \widehat\lambda^X_{N_X})$ and ${\widehat\Lambda}_{N_U}^{U} = \textrm{diag}(\widehat\lambda^U_1, \dots, \widehat\lambda^U_{N_U})$ and $\widehat{\Phi}_{N_X}^{X}$ and $\widehat{\Phi}_{N_U}^{U}$ denote the truncated sets of estimated eigenfunctions or FPCs. However, the joint estimation of $\{{{\xi}}_{i}^{X}, {{\xi}}_{ij }^{U}\}$ can be computationally quite expensive due to the larger dimensions on the second level. Because we only need $\widehat \xi^X$ in the principal component regressions, we use the following expression for the separate estimation of the first-level BLUP proposed by Crainiceanu *et al* (2009) as a simplification of the original PACE BLUP (see Yao *et al* 2005)^6^ 6We estimate the second-level scores only for illustrative purposes (see figure 4), they are not required for the analysis. However, we show the derivation of the corresponding separate second-level estimator in appendix.:

\begin{eqnarray*} \widehat\xi^X_{ik} = \int_0^1 \widehat\lambda_k\widehat\phi_{k}^X\left(t\right)\left[\left(\widehat R_X + \widehat\sigma_{\varepsilon_X}^2\textrm{I}_p\right) + \left( \widehat R_U + \widehat\sigma_{\varepsilon_U}^2 \textrm{I}_p\right)/J \right]^{-1}\widehat{Y_i^X}\left(t\right) \mathrm{d}t,\end{eqnarray*}

where $\widehat \sigma^2_\varepsilon = \widehat\sigma_{\varepsilon_X}^2+\widehat\sigma_{\varepsilon_U}^2$, and $\widehat R_U$ and $\widehat R_X$ are the level-specific smoothed covariance operators. $\widehat{Y^X} = \sqrt{IJ}$ $\left(Y G_X^{0.5}\right)$ is the subject-specific separated data matrix and approximates the latent processes $X_i(t)$.^7^ 7Note that, $\sqrt{IJ}\left(YG_X^{0.5}\right)$ is of dimension *p* × *IJ*. However, the *p* × *I* dimensional matrix of the separated $X-$level data is repeated *J* times so that $\widehat{Y^X}$ corresponds just to the first *I* columns of $\sqrt{IJ}\left(YG_X^{0.5}\right)$.
*G_X_* is the level- and sampling design-specific matrix facilitating the estimation of the covariance operator *K_X_* and is defined in the next section.

### 3.2. Level separation—estimation of the covariance operators

For performing level-specific spectral or eigendecomposition to obtain eigenvalues and eigenfunctions, the level-specific covariance operators $K_X = \mathbb{E}\left[X_i(s), X_i(t)\right]$ and $K_U = \mathbb{E}\left[U_{ij}(s), U_{ij}(t)\right]$ are crucial. A specific feature of SFPCA is to estimate *K_X_* and *K_U_* via the symmetric MoM approach by Koch (1968) adapted to the functional context by Shou *et al* (2015) to create unbiased level-specific covariance matrices of dimension *p* × *p*. The covariance operators for each of the latent processes take a ‘sandwich’ form, $\widehat{K}_X(t,s) = {{Y}}{{G}}_X {{Y}}^{^{\prime}}$ and $\widehat{K}_U(t,s) = {{Y}}{{G}}_U {{Y}}^{^{\prime}}$, where *Y* is assumed to be demeaned. ${{G}}_X$ and ${{G}}_U$ are sampling design-specific sparse *IJ* × *IJ* matrices. For the two-way functional nested model, these matrices equal^8^ 8The representation is derived from the data example in Xiao *et al* (2016), section 7 and appendix.:

\begin{eqnarray*} \begin{gathered} \begin{split} {G}_X &amp; = \frac{1}{IJ \left(J-1\right)} \begin{pmatrix} {0}_I &amp; \dots &amp; \dots&amp;{\mathcal{I}}_I&amp;\mathcal{I}_I\\ \mathcal{I}_I &amp; {0}_I &amp; \dots&amp; \mathcal{I}_I&amp; \mathcal{I}_I\\ \vdots &amp;\dots &amp; \ddots&amp; \dots&amp; \vdots\\ \mathcal{I}_I &amp; \mathcal{I}_I &amp;\dots&amp; \ddots&amp;\mathcal{I}_I\\ \mathcal{I}_I&amp; \dots &amp; \dots&amp; \dots&amp;{0}_I \end{pmatrix},\\ {G}_U &amp; = \frac{1}{IJ \left(J-1\right)} \begin{pmatrix} \left(J-1\right)\mathcal{I}_I &amp; \dots &amp; \dots&amp;-\mathcal{I}_I&amp;-\mathcal{I}_I\\ -\mathcal{I}_I &amp; \left(J-1\right)\mathcal{I}_I &amp; \dots&amp; -\mathcal{I}_I&amp; -\mathcal{I}_I\\ \vdots &amp;\dots &amp; \ddots&amp; \dots&amp; \vdots\\ -\mathcal{I}_I &amp; -\mathcal{I}_I &amp;\dots&amp; \ddots&amp;-\mathcal{I}_I\\ -\mathcal{I}_I&amp; \dots &amp; \dots&amp; \dots&amp;\left(J-1\right)\mathcal{I}_I \end{pmatrix}. \end{split} \end{gathered}\end{eqnarray*}

By estimating the covariance matrices as shown above, the data is separated *implicitly*, i.e. only the covariance matrices are separated into level-specific operators without *explicitly* estimating the level-specific data matrices. To obtain the separated data for equation (11), we follow Shou *et al* (2015) and estimate them as $YG_X^{0.5}$ and $YG_U^{0.5}$ with an additional normalization (see footnote 5 and appendix)^9^ 9Separating *Y* by means of the matrices in equation (12) and normalizing the result appropriately is tantamount to estimating the level-specific data matrices explicitly, i.e. $\sqrt{IJ}\left(YG_X^{0.5}\right)_i = \frac{1}{J} \sum_{j = 1}^{J} {Y}_{ij}$ and $\sqrt{I(J-1)}\left(YG_U^{0.5}\right)_{ij} = Y_{ij}-\widehat {Y^X_i}$.,

\begin{eqnarray*} \begin{gathered} \begin{split} \widehat{Y^X_i} &amp; = \sqrt{IJ}\left(YG_X^{0.5}\right)_i, \\ \widehat{Y^U_{ij}} &amp; = \sqrt{I\left(J-1\right)}\left(YG_U^{0.5}\right)_{ij}. \end{split} \end{gathered}\end{eqnarray*}

The data matrices in equation (13) represent the subject and the subject-spectrum level that are *incompletely* separated from each other and, thus, the subject-level data matrix still contains subject-spectrum variation. $YG_X^{0.5}$ and $YG_U^{0.5}$, on the other hand, constitute *complete* level separation where the subject-level data matrix does not include any variation of the second level. As a consequence, the covariance of the incompletely separated data is greater than that for the completely separated data,

\begin{eqnarray*} \textrm{Cov}\left(\widehat{Y^X}\right) = \left(\widehat R_X + \widehat\sigma_{\varepsilon_X}^2\textrm{I}_p\right) + \left( \widehat R_U + \widehat\sigma_{\varepsilon_U}^2 \textrm{I}_p\right)/J\end{eqnarray*}

where $(\widehat R_X + \widehat\sigma_{\varepsilon_X}^2\textrm{I}_p)$ is the appropriate subject-level variability (see Crainiceanu *et al* 2009, Di *et al* 2009)^10^ 10Conversely, the covariance for $\widehat{Y^U}$ is smaller than for the subject-spectrum level data, see appendix.. For the separate estimation of the subject level scores in equation (11), the incompletely separated data $\widehat{Y^X}$ is used which is why the covariance has to be adjusted accordingly. As $\widehat{Y^X}$ is a consistent estimator for *X_i_* and *J* = 46 in the data application, the difference is however not substantial^11^ 11If the functional data is not high-dimensional (grid size $p < 10\,000$), Shou *et al* (2015) suggest to smooth the off-diagonal entries of $\widehat K_X$ and $\widehat K_U$ to account for noise in the observed data. In case of high-dimensional functional data, however, Shou *et al* (2015) recommend to apply their suggested rank preserving procedure for reducing the dimension *p* and then smooth the *data* before applying SFPCA. This is because the mapping of eigenvalues or FP scores between the high-dimensional and the reduced dimensional model is no longer one-to-one after first applying the dimension-reducing procedure and then smoothing the covariance matrix in this space..

### 3.3. Outcome regression models

To analyze associations between the subject-level walking features and several physical performance indicators, such as body mass index (BMI), we consider functional regression models of the following form (see Di *et al* 2009)

\begin{eqnarray*} \mathbb{E}\left[Z_i\right] = \beta_0 + \int \beta\left(t\right) X_i\left(t\right) \mathrm{d}t + C_i^{^{\prime}}\gamma\end{eqnarray*}

where *Z_i_* is the vector of the subject-specific outcome variables, *X_i_* is (unobserved) subject-specific walking data, and *C_i_* is a vector of additional, potentially confounding covariates such as sex or smoking status, for instance. Equation (15) can be reformulated and thus simplified as

\begin{eqnarray*} \mathbb{E}\left[Z_i\right] = \beta_0 + \sum_{k = 1}^{N_X}\beta_k \phi^{X}_k\left(t\right)\phi^{X^{^{\prime}}}_k\left(t\right) X_i\left(t\right) + C_i^{^{\prime}}\gamma = \beta_0 + \sum_{k = 1}^{N_X} \beta_k \xi_{ik}+ C_i^{^{\prime}}\gamma\end{eqnarray*}

where we have used the orthogonality of the eigenfunction $\phi^X_k(t)$ to (i) replace $\beta(t)$ by $\beta(t) = \sum_{k}^{N_X} \beta_k \phi^X_k(t)$ and (ii) apply the definition of the scores $\xi_{ik} = \phi^X_k(t)X_i(t)$. Equation (16) shows that (S)FPCA facilitates a dimension reduction in the regression models as well: Instead of the estimated subject-level data, the lower dimensional PC scores are included as proxies for the walking data. They can be considered coordinates of the subject-specific trajectories in the lower dimensional space spanned by the subject-specific walking features.

## 4. Data application—DECOS

In the following, the techniques reviewed in the previous section are used to extract the walking features from the preprocessed DECOS data described in section 2. The corresponding two-way functional nested model is given by

\begin{eqnarray*} \texttt{Y}_{ij}\left(t\right) &amp; = &amp; \mu\left(t\right) + \texttt{subject}_i\left(t\right) + {\texttt{spectrum}}_{ij}\left(t\right) + \varepsilon_{ij}\left(t\right), \, i = 1,\dots, 48, \, j = 1,\dots, 46, \, t = 0.3,\dots, 5.75,\end{eqnarray*}

where $\texttt{Y}_{ij}(t)$ denotes the vector magnitude for the (aligned) FFT spectrum *j* of subject *i* at *t* in the order domain. Furthermore, $\texttt{subject}_i$ is the subject-specific latent stochastic process corresponding to *X_i_* in equation (4) and $\texttt{spectrum}_{ij}$ is the subject-spectrum specific latent stochastic process corresponding to *U*_*ij*_ in equation (4). As stated in section 2, the order domain axis is sampled in steps of 0.01 creating a grid of *p* = 546 sampling points such that $\texttt{Y}_{ij} = \{\texttt{Y}_{ij}(0.3),\dots, \texttt{Y}_{ij}(5.75) \}^{^{\prime}}_{[1 \times 546]}$ and thus $\texttt{Y} = \{\texttt{Y}_{1,1}, \dots, \texttt{Y}_{1,46}, \dots, \texttt{Y}_{48,1}, \dots, \texttt{Y}_{48,46}\}_{[546 \times (46\cdot48)]}$.

Some of the walking spectra in the dimension reduced space turn out negative for some ranges in the order domain. As vector magnitude is defined to be positive, we apply a square root transformation to the data Y, $\widetilde{\texttt{Y}} = (\texttt{Y})^{0.5}$, before performing SFPCA to guard against this issue. The SFPCA algorithm applied for analyzing the square root transformed walking spectra is presented in algorithm 1.

**Table:** 

**Algorithm 1** SFPCA for squared root transformed walking spectra.
(1) **Center** the square root transformed data $\widetilde{\texttt{Y}}$. (2) **Separate** the covariance operator $K_{\widetilde{\texttt{Y}}}$ into the **level-specific** covariance operators $\widehat K_{\texttt{sub}}$ and $\widehat K_{\texttt{spec}}$ by computing $\widetilde{\texttt{Y}}G_{X}\widetilde{\texttt{Y}}^{^{\prime}}$ and $\widetilde{\texttt{Y}}G_{U}\widetilde{\texttt{Y}}^{^{\prime}}$. (3) Obtain the **smooth versions** of $\widehat K_{\texttt{sub}}$ and $\widehat K_{\texttt{spec}}$ by performing steps (a) to (c). (a) Estimate the noise variances $\widehat\sigma_{\varepsilon_{\texttt{sub}}}^2$ and $\widehat\sigma_{\varepsilon_{\texttt{spec}}}^2$ ($\widehat\sigma_\varepsilon^2 = \widehat\sigma_{\varepsilon_{\texttt{sub}}}^2 + \widehat\sigma_{\varepsilon_{\texttt{spec}}}^2$) via the **second order difference method** \begin{eqnarray*} \widehat\sigma^2_{\varepsilon_{\texttt{sub}}} &amp; = &amp; \frac{\frac{1}{I\left(p-2\right)} \sum_{i = 1}^{I} \sum_{t = 1}^{p-2} \left(\left(\widehat{\widetilde{\texttt{Y}}}^{\texttt{sub}}_{i,t} -\widehat{\widetilde{\texttt{Y}}}^{\texttt{sub}}_{i,t-1}\right) - \left(\widehat{\widetilde{\texttt{Y}}}^{\texttt{sub}}_{i,t-1} - \widehat{\widetilde{\texttt{Y}}}^{\texttt{sub}}_{i,t-2}\right)\right)^2}{{2\cdot \textrm{ord} \choose 2}},\, \textrm{ord} = 2\\ \widehat\sigma^2_{\varepsilon_{\texttt{spec}}} &amp; = &amp; \frac{\frac{1}{IJ\left(p-2\right)} \sum_{i = 1}^{IJ} \sum_{t = 1}^{p-2} \left(\left(\widehat{\widetilde{\texttt{Y}}}^{\texttt{spec}}_{i,t} -\widehat{\widetilde{\texttt{Y}}}^{\texttt{spec}}_{i,t-1}\right) - \left(\widehat{\widetilde{\texttt{Y}}}^{\texttt{spec}}_{i,t-1} - \widehat{\widetilde{\texttt{Y}}}^{\texttt{spec}}_{i,t-2}\right)\right)^2}{{2\cdot \textrm{ord} \choose 2}}\end{eqnarray*} with $\widehat{\widetilde{\texttt{Y}}}^{\texttt{sub}} = \sqrt{IJ}\left(\widetilde{\texttt{Y}}G_{X}^{0.5}\right)$ and $\widehat{\widetilde{\texttt{Y}}}^{\texttt{spec}} = \sqrt{I(J-1)}\left(\widetilde{\texttt{Y}}G_{U}^{0.5}\right)$. (b) **Subtract** the level-specific **noise variance from the diagonal elements** of the level-specific covariance operators \begin{eqnarray*} \widetilde K_{\texttt{sub}} = \widehat{K}_{\texttt{sub}} - \widehat\sigma_{\varepsilon_{\texttt{sub}}}^2\textrm{I}_p ,\quad \widetilde K_{\texttt{spec}} = \widehat{K}_{\texttt{spec}} - \widehat\sigma_{\varepsilon_{\texttt{spec}}}^2\textrm{I}_p\end{eqnarray*} (c) Apply Xiao *et al* (2013)’s **sandwich smoother** to $\widetilde K_{\texttt{sub}}$ and $\widetilde K_{\texttt{spec}}$ ^12^. \begin{eqnarray*} \widehat R_{\texttt{sub}} = S_X\widetilde K_{\texttt{sub}}S_X ,\quad \widehat R_{\texttt{spec}} = S_U \widetilde K_{\texttt{spec}}S_U\end{eqnarray*} where *S_X_* and *S_U_* are level-specific smoother matrices.
(4) Perform spectral decomposition/**eigendecomposition** on the smooth covariance operators $\widehat R_{\texttt{sub}}$ and $\widehat R_{\texttt{spec}}$ and obtain **level-specific eigenvalues** $\widehat\lambda_k^{\texttt{sub}}$ and $\widehat\lambda_\ell^{\texttt{spec}}$ and **eigenfunctions** $\widehat\phi_k^{\texttt{sub}}$ and $\widehat\phi_\ell^{\texttt{spec}}$, $k = \ell = 1, \dots, 546$ (5) **Choose $N_\texttt{sub}$ and $N_\texttt{spec}$** by applying CPV threshold rule in equation (9) with *q* = 0.85. (6) Using equation (11), separately **estimate the BLUPs of the subject-specific scores** $\xi_{ik}^{\texttt{sub}}$.

12 Originally, the procedure by Xiao *et al* (2013) was not designed for smoothing covariance matrices. Therefore, (i) we need to perform step (3b) to remove the noise variance and (ii) in case $\widehat R_{l}$, $l \in \{\texttt{sub}, \texttt{spec}\}$ is not symmetric, we replace it by $\widehat R_{l} = \left(\widehat R_{l}+\widehat R_{l}^{^{\prime}}\right)/2$.

**Figure 3.**
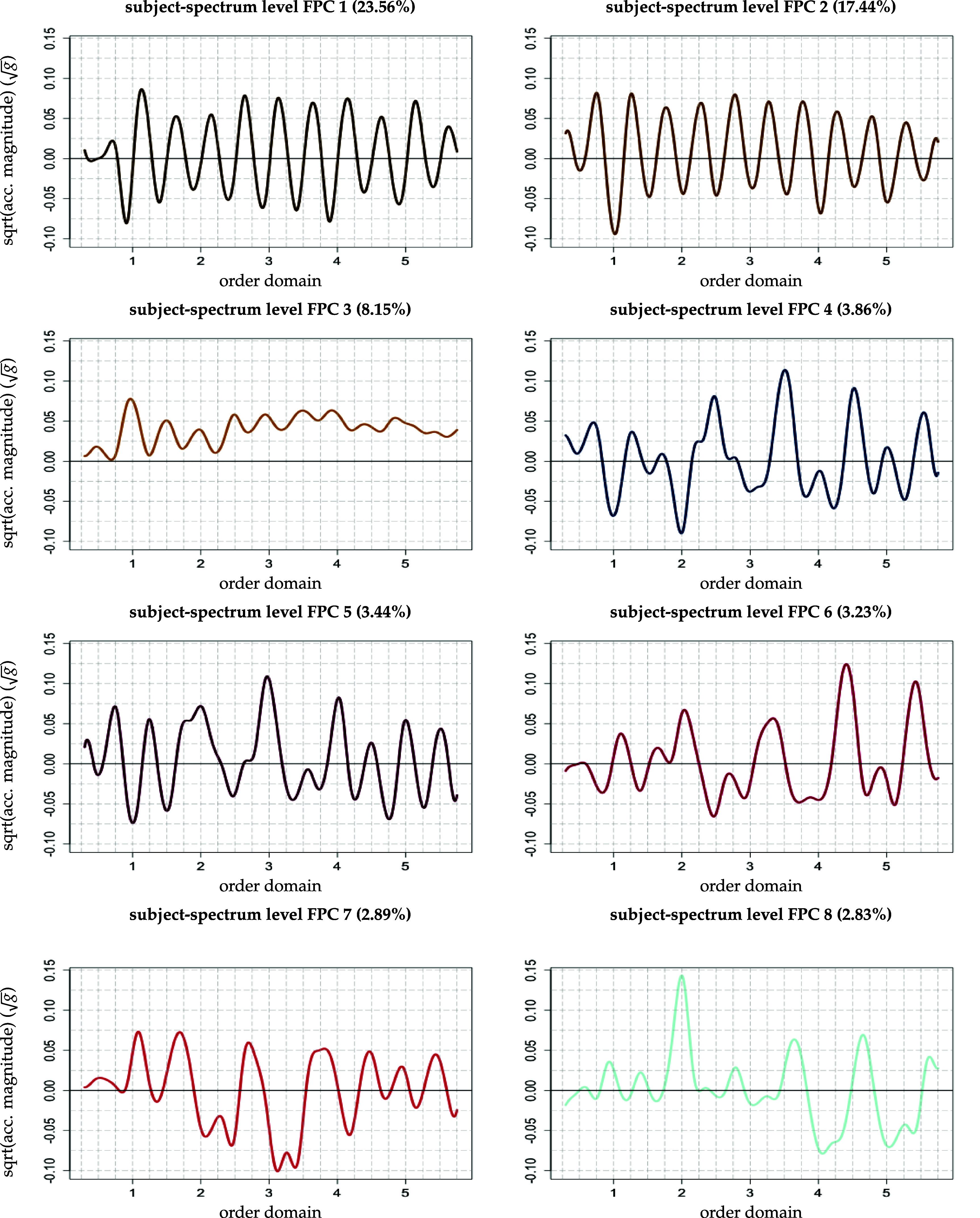
Eight of 19 subject-spectrum level functional principal components of the square root transformed spectra (corresponding proportion of explained total variance in parentheses) Plots start at 0.3 and end at 5.75 in the order domain.

In the following analysis, $\left(\widehat\phi_k^{\texttt{sub}},\widehat\phi_\ell^{\texttt{spec}}\right)$ and $\left(\widehat\xi_{ik}^{\texttt{sub}},\widehat\xi_{ij\ell}^{\texttt{spec}}\right)$ refer to the level-specific eigenfunctions and scores of the *square root transformed* walking spectra. We adapt the rule in (9) with *q* = 0.85, a common choice for the cumulative explained variance (see, e.g. Li *et al* 2015) to capture at least 85% of the level-specific variability of the square root transformed spectra. This is achieved by retaining the first five eigenfunctions on the subject level (figure 2) and the first 19 eigenfunctions of the subject-spectrum level (figure 3). Using this truncated set of eigenfunctions (FPCs) and the associated set of EBLUPs of the FP scores on both levels, model (17) can be approximated by

\begin{eqnarray*} {\widetilde{\texttt{Y}}}_{i j}^{\textrm{KL}}\left(t\right) = \mu\left(t\right) + \sum_{k = 1}^{5} \widehat\phi_{k}^{\texttt{sub}}\left(t\right) \widehat\xi_{i k}^{\texttt{sub}}+\sum_{\ell = 1}^{19} \widehat\phi_{\ell}^{\texttt{spec}}\left(t\right) \widehat\xi_{ij \ell}^{\texttt{spec}} + \varepsilon_{ij}\left(t\right).\end{eqnarray*}

**Figure 4.**
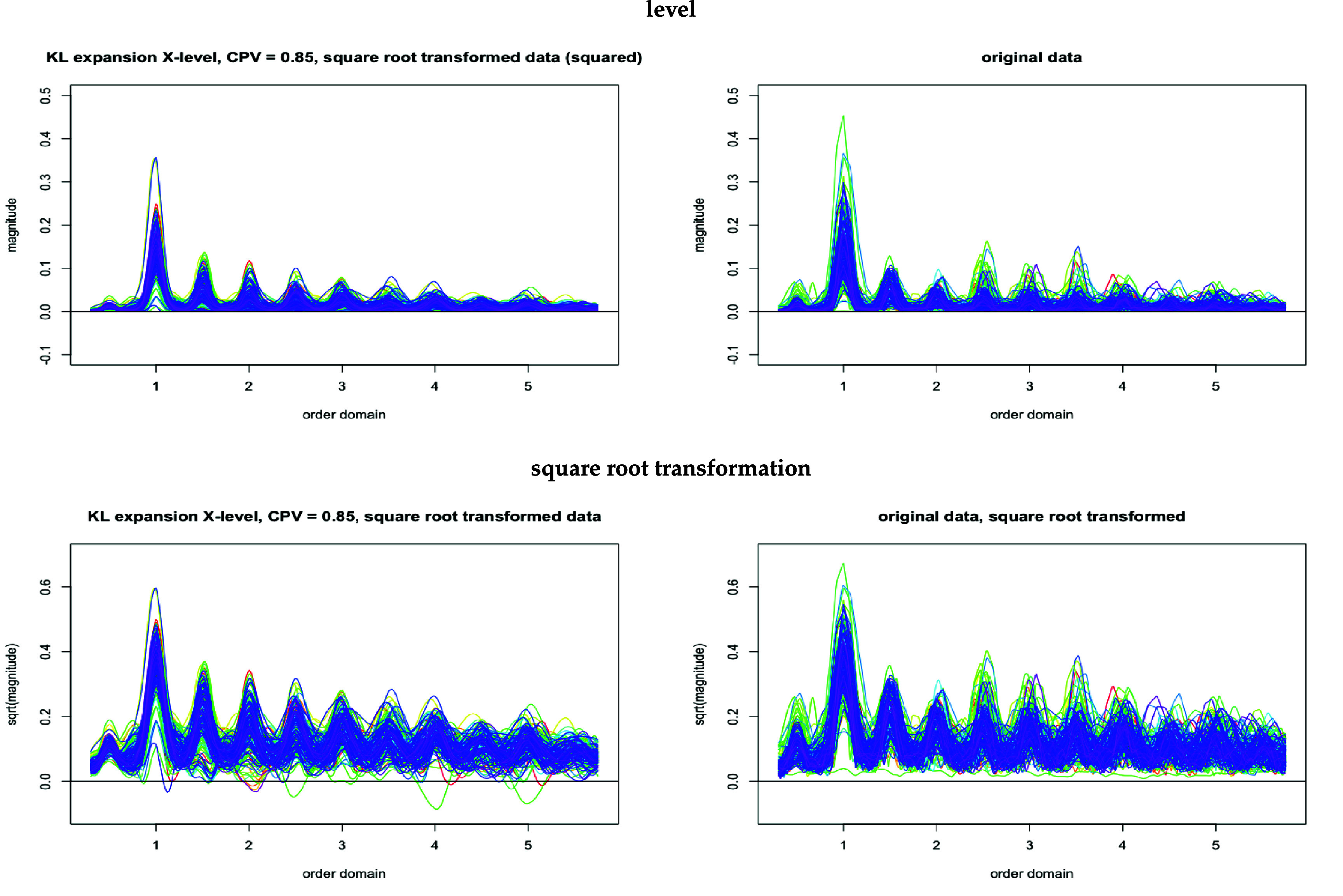
Karhunen-Loève expansion vs. original data (restricted), 221 (= 10%) randomly selected curves.

Figure 4 compares the approximated and the complete data, $\widetilde{\texttt{Y}}^{\textrm{KL}}$ and $\widetilde{\texttt{Y}}$. The upper two graphs show the complete original data on the right and, on the left, the Karhunen-Loève approximation (CPV = 85%) of the transformed data but squared to match it with the original data. The lower two graphs present the complete transformed data on the right and, on the left, again the Karhunen-Loève approximation (CPV = 85%) of the transformed data. Comparing left and right graphs shows that, although the dimensionality of the data has been reduced substantially, the overall shape as well as the level of the curves has been retained. Also, the dimension-reduced data in the left graphs display the harmonics at the cadence and its multiples more clearly than the right graphs do for the complete data. A measure for the variation in square root acceleration magnitude (henceforth: acceleration magnitude) explained by the subject level is the functional counterpart of the *intraclass correlation coefficient* (ICC) known from the mixed model framework (see Di *et al* 2009),

\begin{eqnarray*} {\textrm{ICC}} = \frac{\sum_{k = 1}^{\infty} \lambda_k} {\sum_{k = 1}^{\infty} \lambda_k+\sum_{\ell = 1}^{\infty} \lambda_\ell} \Rightarrow\widehat{\textrm{ICC}} = \frac{\sum_{k = 1}^{5} \lambda_k^{\texttt{sub}}}{\sum_{k = 1}^{5} \lambda_k^{\texttt{sub}}+\sum_{\ell = 1}^{19} \lambda_\ell^{\texttt{spec}}}.\end{eqnarray*}

In mixed model terminology, the ICC specifies the proportion of variance explained by the *within cluster variability*. Here, the cluster is one participant, subject_*i*_, and the spectra, $\texttt{spectrum}_{ij}$, nested within subject_*i*_ are the clustered data. For the acceleration magnitude, we find that $\widehat{\textrm{ICC}} = 0.591$ which means that two spectra belonging to the same participant are positively correlated.

**Figure 5.**
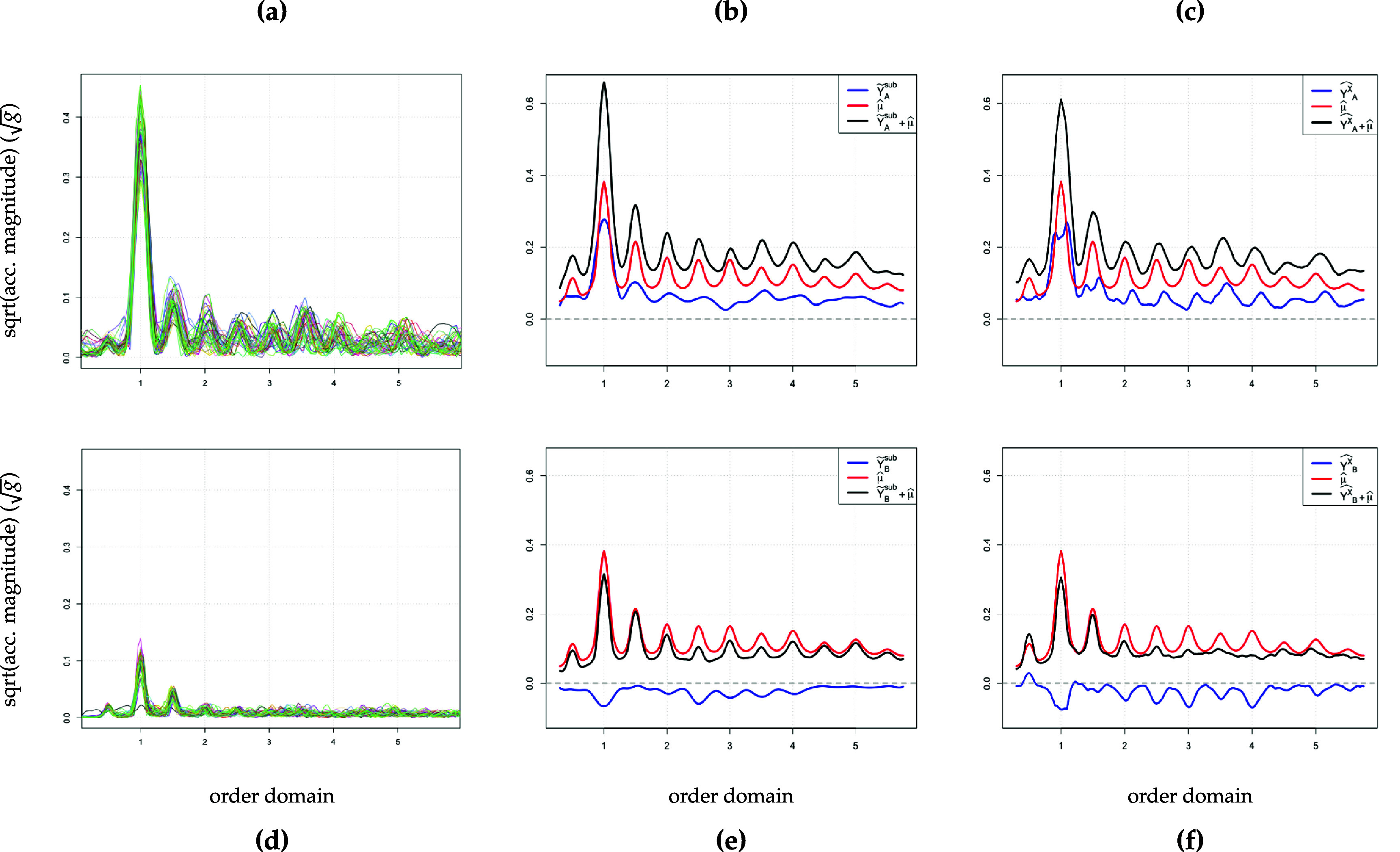
Aligned FFT spectra and walking pattern (vs. separated data) for two participants. Panel (a) realigned spectra, (b) walking pattern by features, (c) walking pattern by separated data for Participant A. Panel (d) realigned spectra, (e) walking pattern by features, (f) walking pattern by separated data for Participant B.

The level-specific FPCs can be considered walking features extracted on the respective level. Thus, the linear combination of the subject-specific walking features weighted by their scores, ${\widetilde{\texttt{Y}}}^{\texttt{sub}}(t) = \sum_{k = 1}^{5} \widehat\phi_{k}^{\texttt{sub}}(t) \widehat\xi_{k}^{\texttt{sub}}$, an approximation for the subject-level random process $\texttt{subject}(t)$. ${\widetilde{\texttt{Y}}}^{\texttt{sub}}(t)$ represents the functional correspondent to a *between-subject random effect* and hence models the (random) deviation of subject *i*’s walking pattern (averaged across all spectrum curves) in terms of acceleration magnitude from the average walking pattern of all participants (fixed effect). Thus, $\widetilde{\texttt{Y}}_{i}^{\texttt{sub}}$ can be negative as well as positive as displayed in figure 5, plots (b) versus (e). Together with the fixed effect $\widehat{\mu}(t)$, $\widetilde{\texttt{Y}}_{i}^{\texttt{sub}}$ produces the average walking acceleration magnitude for subject *i* which can be considered the subject-specific *walking pattern* $\textrm{wp}_i$,

\begin{eqnarray*} {\textrm{wp}_i\left(t\right) } = \widehat{\mu}\left(t\right) + {\widetilde{\texttt{Y}}}_{i}^{\texttt{sub}}\left(t\right).\end{eqnarray*}

For each participant, the walking pattern is thus summarized in one curve which is adjusted for redundancies and noise. In figure 5, we again display the aligned FFT spectra curves for participant A and B from figure 1 together with their associated walking pattern and subject-specific deviation given by the weighted sum of the walking features $\widehat \phi_k^X$. Superficially, ${\widetilde{\texttt{Y}}}_{i}^{\texttt{sub}}$ seems to be similar to $\widehat{Y^X_i}$ in equation (13), an approximation of the unobserved subject-specific curve $\texttt{subject}_i$. However, compared to $\widetilde{\texttt{Y}}_{i}^{\texttt{sub}}$, $\widehat{Y^X_i}$ is a quite crude estimate of the subject-specific deviations without the potential that (M)FPCA offers by providing five individual features that can serve as predictors in further analyses.

A measure for a feature’s importance within the subject-specific walking patterns is the percentage of level-specific variability explained by the specific feature. This is because this quantity provides information about how much of the heterogeneity (as subject-specific deviation from the population mean) between participants is captured by each feature. While, cumulatively, the five features explain about 86.5% of the subject-level variability, roughly 60% is explained solely by the first walking feature. This finding indicates that most of the heterogeneity between the participants is captured by this walking feature alone. As the first feature’s most pronounced peak occurs at the cadence, the prior result suggests that participants vary most strongly by their acceleration magnitude at the cadence. The second most important walking feature contributes about 11% to the explanation of the subject-level variability and the third approximately 7% leaving less than 9% for the two remaining features.

The pronounced peaks and valleys^13^ 13We consider peaks and valleys ${\unicode{x2A7E}}|0.1|$ to be pronounced. at the cadence and its multiples in the individual features can be interpreted in terms of acceleration magnitude given the sign of a participant’s score. The peak at the cadence in the first walking feature tells us that subject *i* with higher score $\xi_{1i}^{\texttt{sub}} > 0$ has a higher acceleration magnitude at the cadence than the average participant. As another example, consider the second feature. Subject *i* with higher score $\xi_{2i}^{\texttt{sub}} > 0$ has a lower acceleration magnitude at the cadence and a higher acceleration magnitude at cadence multiples 2.5 and 3.5 than the average participant. The same reasoning applies to the remaining three features.

Following the standard linear mixed effects model interpretation, the subject-spectrum-specific features constitute, broadly speaking, the *random spectrum-specific deviations* from the subject-specific walking pattern generated by the first-level walking features and scores and the fixed effect (see equation (20)). Due to their periodic nature, their individual interpretation is more complicated.

We find that in terms of overall data variability, the subject-level effects account for about 54% compared to about 46% explained by the subject-spectrum-specific effects. This indicates that, from one several-second time window to the next, the walk of *the same* participant is somewhat less variable than the walks of *different* participants compared to each other.

The subject-level scores are further used in linear regression models as specified in equation (16) to study the association between the components of the individual walking pattern and health indicators. For a given set of included scores, we use the Wald test for the null hypothesis of a regression coefficient equal to zero. Initially, we regress an outcome variable on all five subject-level scores as well as the additional regressors Age and Sex:

\begin{eqnarray*} {\mathbb{E}}\left[\texttt{outcome}_i\right] = \beta_{0} + \sum_{k = 1}^5 \widehat{\xi}_{ik}^\texttt{sub} \beta_{k} + {\texttt{Sex}_i} \cdot \gamma_{1}+ \texttt{Age}_i \cdot \gamma_{2} ,\end{eqnarray*}

where outcome is one of {Age, BMI, BPM, Avg_cadence, PFS_mental ^14^ 14The abbreviation PFS refers to the ten-item *Pittsburgh Fatigability Scale* (see Glynn *et al* 2015) which is a tool to evaluate fatigability in older adults., PFS_physical MAP, SPPB}. The outcome variables are defined and explained in table 1. After dropping participants with missing observations, we end up with a sample size of 45 participants. The regression results are displayed in table 2.

**Table 1.** Outcome variables.

Abbreviation	Outcome	Definition
BMI	Body mass index	kg m^−2^
BPM	Heart rate	beats min^−1^
Avg_cadence	Average cadence	averaged across 400 m walk, steps s^−1^
PFS_mental	Fatigability, mental	10 item mental scores, range 0–50
PFS_physical	Fatigability, physical	10 item physical scores, range 0–50
Sex	Sex	female = 0, male = 1
MAP	Mean arterial pressure	Avg. pressure in participant’s arteries
		during one cardiac cycle
		MAP = (syst. BP + 2×diast. BP)/3
SPPB	Short physical performance battery	Measure of physical function
		score range 0–12

**Table 2.** Coefficients and *p*-values (second line in parentheses, highlighted in gray if $ {\lt} 0.05$) for final models selected via BIC. Percentage of explained variability on subject level per feature given in parentheses in left column. Percentage of variation of outcome variables explained by forced-in confounders Age and Sex given by $R^2_{\textrm{adj, basic}}$. Percentage of variation of outcome variables explained by scores and confounders Age and Sex given by $R^2_{\textrm{adj, full}}$. (*I*_reg_ refers to the number of observations available for the respective regression model.)

$({\beta_0}, {\gamma}, {\beta_k})$/*Z*	Age	BMI	BPM	Avg_cad	PFS_mental	PFS_physical	MAP	SPPB
Const	78.413	53.007	88.948	1.769	12.735	10.000	126.366	16.764
Age		−0.342	−0.311	0.003	−0.063	0.080	−0.442	−0.078
		(0.003)	(0.298)	(0.468)	(0.817)	(0.709)	(0.165)	(0.118)
Sex	1.065	0.998	−4.407	−0.067	1.034	−1.383	−6.278	−0.447
	(0.464)	(0.368)	(0.124)	(0.163)	(0.701)	(0.506)	(0.074)	(0.343)
${\widehat\xi_1}$	−5.691	−3.747	−3.509	0.194	−5.054	−3.654		1.234
(59.67%)	($ < $0.001)	(0.002)	(0.272)	($ < $0.001)	(0.087)	(0.113)		(0.023)
${\widehat\xi_2}$	6.915						16.696	
(10.82%)	(0.017)						(0.035)	
${\widehat\xi_3}$	8.812	−7.349		−0.252				
(7.23%)	(0.026)	(0.021)		(0.063)				
${\widehat\xi_4}$	12.585							
(5.03%)	(0.007)							
${\widehat\xi_5}$								
(3.79%)								
R$^2_{\textrm{adj, basic}}$	−0.023	0.048	0.046	0.077	−0.030	0.048	0.038	0.192
R$^2_{\textrm{adj, full}}$	0.474	0.324	0.051	0.325	0.027	0.088	0.116	0.272
I_reg_	45	45	45	45	39	40	45	45

The binary variable Sex (male = 1, female = 0) is the only additional regressor when outcome = Age. The optimal subset regression models are searched across all possible subsets with Age and Sex forced in. We use the Bayesian Information Criterion (BIC) as selection criterion for the exhaustive search approach which yields the models given in table 2. In the table cells, the numbers in the first line indicate the coefficient value $\widehat\beta_k^{\texttt{outcome}}$ while the numbers in parentheses highlighted in gray in the second line indicate the associated *p*-values. We only discuss those results for which the estimated coefficients are significant at 5%.

**Figure 6.**
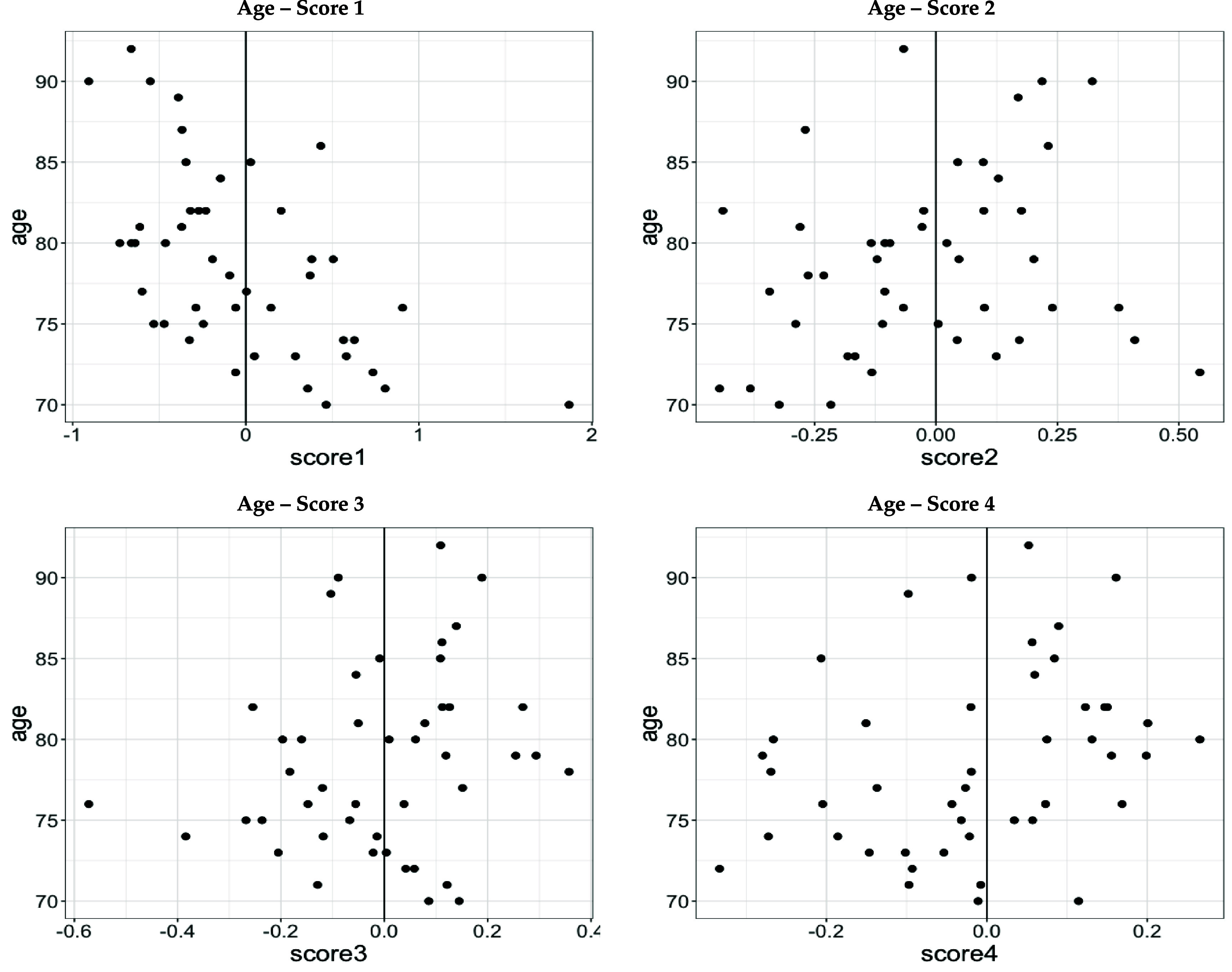
Scatter plots of subject level functional principal scores and age.

**Figure 7.**
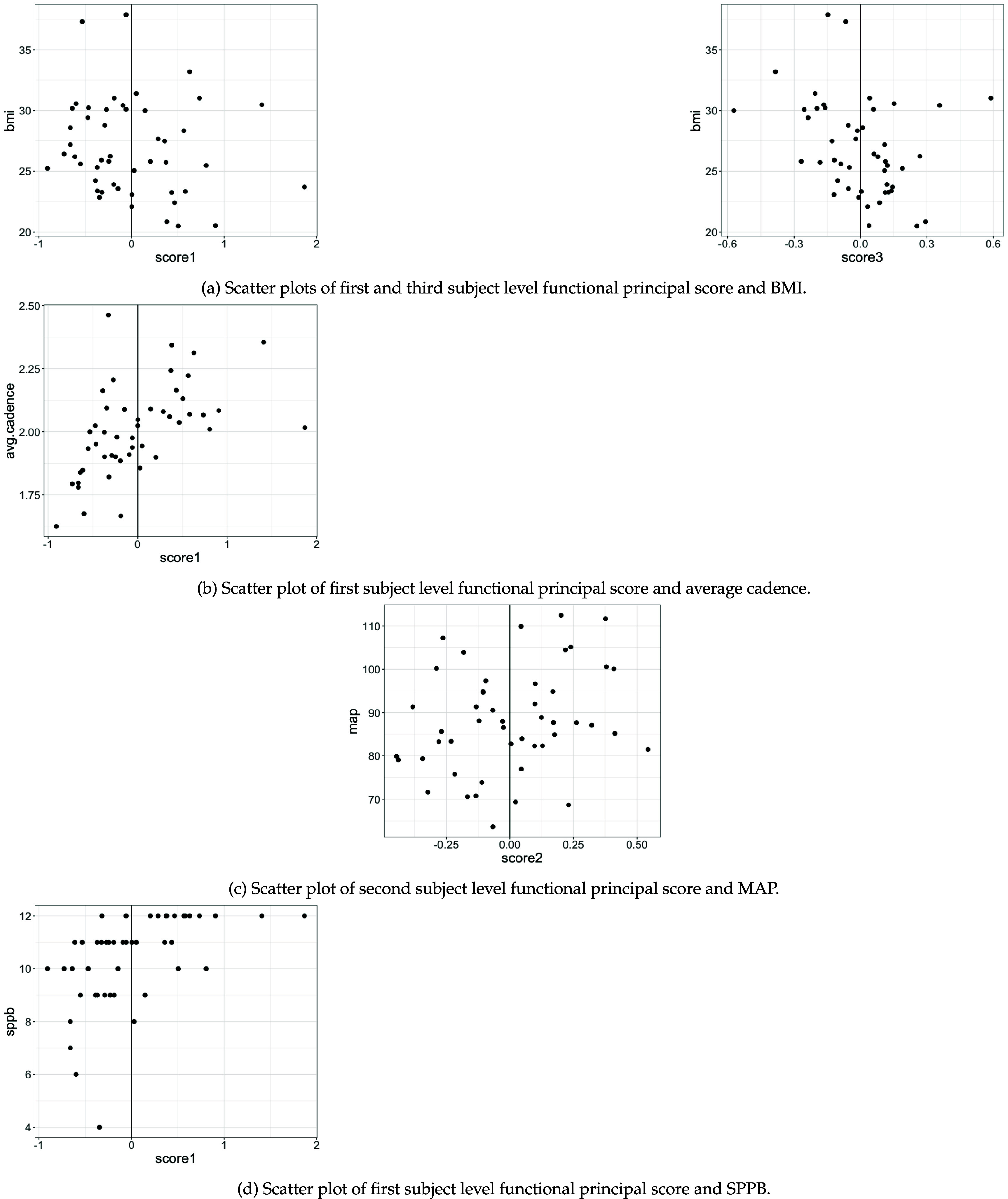
Scatter plots of subject level functional principal scores and health outcomes.

For clarity of interpretation, assume $\widehat{\xi}_{ik}^\texttt{sub} > 0$. For $\widehat{\xi}_{ik}^\texttt{sub} < 0$ the reverse applies. There are two mechanisms, linked through the scores, that enable us to relate the acceleration magnitude generated by the *k*th walking feature with the ‘outcome—score’–association obtained from the regression models. First, the scores $\widehat{\xi}_{ik}^\texttt{sub}$ can be positively or negatively associated with the outcomes. In figures 6 and 7 we additionally provide plots for the significant associations between the scores and the health outcomes where each dot corresponds to one participant. For example, in figure 6, top right graph, the strongly negative relationship between age and the first score is clearly visible: Older participants tend to have smaller, even negative, values for score 1. Second, the peaks and valleys at the cadence and its multiples of the separate walking features multiplied with the associated scores indicate whether a participant has a higher or lower acceleration magnitude (compared to the average participant) at the respective harmonic in the order domain. Based on these two considerations, we can make an attempt at predicting the direction of the outcome variable given the acceleration magnitude at the (qualitatively) larger harmonics of the walking features. In the following paragraphs, we offer an interpretation of the results in table 2 in connection with the walking features in figure 2 ^15^ 15As an example, we display a more detailed interpretation for age as the health outcome in section 4.1..

The health indicators age, BMI, average cadence and SPPB are significantly related to the first walking feature proxied for by the the first score $\widehat{\xi}_{i1}^\texttt{sub}$. Thus, higher $\widehat{\xi}_{i1}^\texttt{sub} > 0$ are associated with

– younger age ($\widehat\beta_1^{\texttt{age}} = -5.69$, *p*-value $\lt$ 0.001),
– lower BMI ($\widehat\beta_1^{\texttt{bmi}} = -3.75$, *p*-value = 0.002),
– faster average cadence ($\widehat\beta_1^{\texttt{cad}} = 0.19$, *p*-value $\lt$ 0.001),
– and higher SPPB scores ($\widehat\beta_1^{\texttt{sppb}} = 1.234$, *p*-value = 0.023).

The first walking feature displays a pronounced peak at the cadence indicating that participants with $\widehat{\xi}_{i1}^\texttt{sub} > 0$ have a higher acceleration magnitude at the cadence. Taking these two observations together and holding everything else fixed leads to the following finding: Participants who have a higher acceleration magnitude at the cadence, are predicted to be younger, have a lower BMI, make more steps per second, and have a better physical function than the average participant.

The second score is selected as explanatory variable into the models for age and mean arterial pressure (MAP). Higher second scores $\widehat{\xi}_{i2}^\texttt{sub}$ are associated with older age ($\widehat\beta_2^{\texttt{age}} = 6.92$, *p*-value = 0.017) and higher MAP ($\widehat\beta_2^{\texttt{map}} = 16.70$, *p*-value = 0.035). The corresponding second walking feature $\widehat\phi_2^{\textrm{sub}}$ exhibits a valley at the cadence and a peak at cadence multiples 2.5 and 3.5 suggesting that participants with with $\widehat{\xi}_{i2}^\texttt{sub} > 0$ have a lower acceleration magnitude at the cadence and higher acceleration magnitude at cadence multiples 2.5 and 3.5 than the average participant. Hence, participants displaying this acceleration magnitude pattern are anticipated to be older and have a higher MAP.

The third score is selected into the models for age and BMI. Based on the estimated coefficients, higher third scores $\widehat{\xi}_{i3}^\texttt{sub}$ are associated with older age ($\widehat\beta_3^{\texttt{age}} = 8.81$, *p*-value = 0.026) and lower BMI ($\widehat\beta_3^{\texttt{bmi}} = -7.35$, *p*-value = 0.021). The third walking feature $\widehat\phi_3^{\textrm{sub}}$ has one pronounced valley at cadence multiple 2.5. Thus, participants with $\widehat{\xi}_{i2}^\texttt{sub} > 0$ who will show a lower acceleration magnitude at this cadence multiple are predicted to be older, and to have a lower BMI.

Age and the fourth score are significantly associated ($\widehat\beta_4^{\texttt{age}} = 12.59$, *p*-value = 0.007). Participants having a lower acceleration magnitude at cadence multiple 1.5, where the fourth walking feature displays a pronounced valley, are predicted to be older than the average participant. The fifth score is not selected into any model.

### 4.1. Interpretation example for outcome variable Age

**Figure 8.**
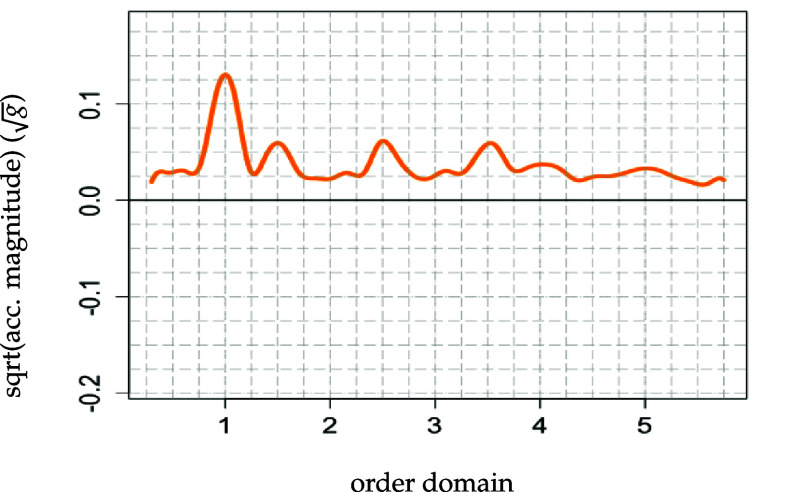
First subject-specific level functional principal component of the square root transformed spectra. Plot starts at 0.3 and ends at 5.75 in the order domain.

(1)
**For participant *i* with $\widehat{\xi}_{i1}^\texttt{sub} > 0$**
  \begin{eqnarray*} &amp;&amp;\underbrace{\widehat{\xi}_{i1}^\texttt{sub}}_{{ > 0}} \times \underbrace{\widehat\phi^{\texttt{sub}}_1\left(t\right)}_{\tiny{\begin{array}{c} > \ \textrm{at cadence } \\ \end{array}}} = {\widetilde{\texttt{Y}}_{i}^{\textrm{feat1}} \left(t\right)}_{} \Rightarrow {{\mathbb{E}}\left[\texttt{Age}_i\right]}_{{}} = \underbrace{\widehat{\xi}_{i1}^\texttt{sub}}_{{ > 0}} \times \underbrace{\widehat \beta_1^{\texttt{age}}}_{\left(-5.69\right)} + \dots\end{eqnarray*}
  (a) **Karhunen-Loève:** peak at cadence^16^ 16
$t = 1 = $ cadence in the order domain corresponding to $p = 101 = $ grid point. of $\widehat\phi^{\texttt{sub}}_1(t)$ (displayed in figure 8 above) multiplied with $\widehat{\xi}_{i1}^\texttt{sub}$
    → participant *i* with $\widehat{\xi}_{i1}^\texttt{sub} > 0$ has **higher acceleration magnitude, $\widetilde{\texttt{Y}}_{i}^{\textrm{feat1}} (t)$, at the cadence** (*t* = 1, *p* = 101) in order domain compared to average participant
  (b) **Outcome regression:** $\widehat{\xi}_{i1}^\texttt{sub}$ is negatively associated with age, $\widehat \beta_1^{\texttt{age}} = -5.69$
    → participant *i* with higher $\widehat{\xi}_{i1}^\texttt{sub} > 0$ is predicted to be **younger**
  (c) **Summary:** **higher acceleration magnitude at cadence may hint at younger age**
(2)
**For participant *i* with $\widehat{\xi}_{i1}^\texttt{sub} < 0$**
  \begin{align*} &amp;&amp;\underbrace{\widehat{\xi}_{i1}^\texttt{sub}}_{{ < 0}} \times \underbrace{\widehat\phi^{\texttt{sub}}_1\left(t\right)}_{\tiny{\begin{array}{c} > \ \textrm{at cadence } \\ \end{array}}} = {\widetilde{\texttt{Y}}_{i}^{\textrm{feat1}} \left(t\right)}_{} \Rightarrow {{\mathbb{E}}\left[\texttt{Age}_i\right]}_{{}} = \underbrace{\widehat{\xi}_{i1}^\texttt{sub}}_{{ < 0}} \times \underbrace{\widehat \beta_1^{\texttt{age}}}_{\left(-5.69\right)} +\dots\end{align*}
  (a) **Karhunen-Loève:** peak at cadence of $\widehat\phi^{\texttt{sub}}_1(t)$ (displayed in figure 8 above) multiplied with $\widehat{\xi}_{i1}^\texttt{sub}$
    → participant *i* with $\widehat{\xi}_{i1}^\texttt{sub} < 0$ has **lower acceleration magnitude, $\widetilde{\texttt{Y}}_{i}^{\textrm{feat1}} (t)$, at the cadence** (*t* = 1, *p* = 101) in order domain compared to average participant
  (b) **Outcome regression:** $\widehat{\xi}_{i1}^\texttt{sub}$ is negatively associated with age, $\widehat \beta_1^{\texttt{age}} = -5.69$
    → participant *i* with lower $\widehat{\xi}_{i1}^\texttt{sub} < 0$ is predicted to be **older**
  (c) **Summary:** **lower acceleration magnitude at cadence may hint at older age**

## 5. Discussion

This paper proposes the extraction of level-specific walking features in the frequency/order domain on two levels via structured FPCA, a multilevel FPCA procedure. The data analyzed is a collection of functional time series created by a preprocessing algorithm out of raw accelerometry data. The distinctive feature of structured FPCA is the level separation (separation of covariance operators) of the hierarchical data achieved through design-specific matrices. Depending on the sampling scheme (crossed and nested) and the number of levels, the level-specific matrices may take different forms.

The new subject and subject-spectrum level features are extracted from walking data representing the fast-paced 400 m corridor walk performed by older adults. The initial high-frequency three-dimensional signal per participant obtained from an accelerometer is transformed to a single functional time series in the frequency domain where the curves are FFT spectra. For that purpose, the preprocessing procedure makes use of the periodicity inherent in the walking movement to reduce noise while preserving individual features. One such characteristic is the subject-specific cadence which facilitates aligning the spectra of all participants for the analysis (transformation to the order domain).

Thus, considering the preprocessed accelerometry data functional data provides us with additional *time-ordered* intra-walk information inherent in the smooth spectrum curves. Not only can we make use of the cadence per spectrum curve to align the walks (intra- and inter-participant) but it can also be used as a proxy-indicator for walking intensity (see Tudor-Locke *et al* 2018). The shape of the spectrum curves can tell even more about a person’s walk. The amount of energy located at the frequencies, that is the pronunciation of the peaks, can quantify the walking asymmetry/smoothness (see Fadel *et al* 2020). If more energy is distributed at higher frequencies, the participant displays a more unstable walk marked by, for example, stumbling or limping. A smoother walking pattern is encountered if more energy is pooled around the cadence.

As a result of the complexity reduction, the walking features on the participant level are more easily interpretable than subject-specific spectra plots. The linear combination of these walking features generates the individual-specific walking pattern. The walking features on the subject-spectrum-specific level constitute the random spectrum-specific deviations from this pattern. We use the scores of the subject-level features as possible predictors for health outcomes including SPPB, BMI, and (physical and mental) fatigability. We find that the subject-level features are significantly related with several indicators of physical health suggesting that the individual walking pattern may shed light on a subject’s subclinical disease status. Especially higher acceleration magnitude at the cadence expressed in the first walking feature seems to be a predictor for relatively better overall health. In particular, this walking feature is strongly associated with lower BMI, faster average cadence and better physical function. This finding potentially indicates that the 400 m corridor walk performance for older adults can act as a prognostic factor for several health outcomes which adds another angle to existing evidence (see, e.g. Newman *et al* 2006). We also show that the first subject-specific walking feature explains most of the variability between participants, i.e. it pools most of the information contained in the subject level.

Our study relies on a relatively small sample (*I* = 48) of healthy elderly individuals. Additionally, the data were collected in a lab environment which lacks certain conditions inherent to the real-world environment such as uneven surfaces, inclines, etc. These two circumstances limit the generalizability of our findings to other and/or extended populations and data collected in a different setting, e.g. in free-living conditions. Furthermore, we only consider one aspect of physical activity in our analysis which does not entirely capture an individual’s movement. Additional information can potentially be obtained from other aspects such as climbing stairs to construct a more holistic movement pattern. In our analysis, we use the cumulative percentage of explained variance threshold with *q* = 0.85. This common choice for the threshold ensures the exclusion of eigenfunctions from the analysis that are cyclical and thus less interpretable. Different values for *q* may lead to a different number of retained eigenfunctions and thus to a different number of walking features. As our analytical method relies on a relatively complex functional regression model, the sample size of our study (*I* = 48) is not optimal for proper comparisons to be made for either the interaction effects by sex or the analysis stratified by sex. We plan to undertake such analyses on raw data collected in a larger NHANES III study (National Center for Health Statistics (NCHS) 2012, 2014).

One aspect for future research could be the transfer of the procedures used in this paper to data collected in free-living conditions. Apart from their shape, it might be interesting to investigate the associations between the walking features obtained in free-living conditions and health indicators and compare these results to those obtained in this paper. Urbanek *et al* (2018) examine both, gait characteristics identified from in-the-lab data and data collected under free-living conditions, and their association with each other as well as with several measures of physical function, mobility, fatigability, and fitness. The walking characteristics used in their paper are obtained via the sustained harmonic walking algorithm by Urbanek *et al* (2018). They furthermore suggest that in a controlled environment the association between walking characteristics and physical fatigability might be downward-biased from the participants being well-rested and completely aware of the study purpose. Data obtained under free-living conditions is less restricted which is why the analysis of such data might also shed more light on the association between the walking features and (mental and physical) fatigability.

In conclusion, we found that walking features extracted from raw accelerometry data may be used to make predictions about an individual’s health status. In the reported work, we investigated the association between the individual-specific walking pattern, which is composed of the extracted features, and several measures of physical performance. By using SFPCA we reduced the complexity of the data and thus made them more accessible for a meaningful analysis. In contrast to previously applied extraction methods, functional PCA accounts for the varying cadence and commonality of features among participants in the sample. Moving forward, walking features might be a useful tool to shed light on an individual’s subclinical health problems.

## Data Availability

The data cannot be made publicly available upon publication because no suitable repository exists for hosting data in this field of study. The data that support the findings of this study are available upon reasonable request from the authors.

## Appendix Variance of the separated $U-$level data

For the sake of legibility we drop the arguments and treat *Y*_*ij*_ as perfectly observed ($\sigma_e^2 = \sigma_\varepsilon^2 = 0$) in the following. The noise variance can be added back to the expressions in a straightforward manner once the results are obtained.

The variance of *Y*_*ij*_ is given as

\begin{eqnarray*} \operatorname{Var}\left(Y_{i j}\right) = \Phi^X \Lambda^X \Phi^{X}+\Phi^U \Lambda^U \Phi^{U^{^{\prime}}}.\end{eqnarray*}

Averaging out the curve-level, we can rewrite the $X-$level data in terms of the $X-$ and $U-$level eigenfunctions and scores

\begin{eqnarray*} \widehat{Y^X_i}&amp; = &amp;\frac{1}{J} \sum_{j = 1}^J Y_{i j} = \Phi^X \xi^X_i+\frac{1}{J} \Phi^U \sum_{j = 1}^J \xi^U_{ij}.\end{eqnarray*}

The variance of the separated $X-$level data is then obtained as

\begin{eqnarray*} \begin{aligned} \operatorname{Var}\left(\widehat{Y^X_i}\right) &amp; = \Phi^X \Lambda^X \Phi^{X^{^{\prime}}}+\frac{1}{J^2} \Phi^U \operatorname{Var}\left(\sum_{j = 1}^J \xi^U_{ij}\right)\Phi^{U ^{^{\prime}}} \\ &amp; = \Phi^X \Lambda^X \Phi^{X^{^{\prime}}}+\frac{1}{J^2} \Phi^U \sum_{j = 1}^J\operatorname{Var}\left( \xi^U_{ij}\right)\Phi^{U ^{^{\prime}}} \\ &amp; = \Phi^X \Lambda^X \Phi^{X^{^{\prime}}}+\frac{1}{J}\Phi^U \Lambda^U \Phi^{U^{^{\prime}}}\\ \end{aligned}\end{eqnarray*}

which is the same result as in Crainiceanu *et al* (2009).

For finding the variance of the separated $U-$level data as given below, we need the covariance of the original data *Y*_*ij*_ and the separated $X-$level data

\begin{eqnarray*} \operatorname{Var}\left(\widehat{Y^U_{ij}}\right) = \operatorname{Var}\left(Y_{ij}-\widehat{Y^X_i}\right) = \operatorname{Var}\left(Y_{i j}\right)+\operatorname{Var}\left(\widehat{Y^X}_i\right)-2 \operatorname{Cov}\left(Y_{i j}, \widehat{Y^X}_i\right).\end{eqnarray*}

The covariance is given as

\begin{eqnarray*} \begin{gathered} \begin{split} \operatorname{Cov}\left(Y_{i j}, \widehat{Y^X_i}\right)&amp; = \operatorname{Cov}\left(Y_{i j}, \frac{1}{J} \sum_{j = 1}^J Y_{i j}\right) \\ &amp; = \operatorname{Cov}\left(\Phi^X \xi^X_i+\Phi^U \xi^U_{ij}, \Phi^X \xi^X_i+ \frac{1}{j} \Phi^U \sum_{j = 1}^J \xi^U_{ij}\right) \\ &amp; = \Phi^X \Lambda^X \Phi^{X^{^{\prime}}}+\frac{1}{J} \Phi^U \operatorname{Cov}\left(\xi^U_{ij}, \sum_{j = 1}^J \xi^U_{ij}\right)\Phi^{U^{^{\prime}}} \\ &amp; = \Phi^X \Lambda^X \Phi^{X}+\frac{1}{J}\Phi^U \Lambda^U \Phi^{U^{^{\prime}}}, \end{split} \end{gathered}\end{eqnarray*}

which leads to

\begin{eqnarray*} \begin{gathered} \begin{split} \operatorname{Var}\left(\widehat{Y^U_{ij}}\right)&amp; = \operatorname{Var}\left(Y_{i j}\right)+\operatorname{Var}\left(\widehat{Y^X_i}\right)-2 \operatorname{Cov}\left(Y_{i j}, \widehat{Y^X_i}\right)\\ &amp; = \cancel{\Phi^X \Lambda^X \Phi^{X}}+\Phi^U \Lambda^U \Phi^{U^{^{\prime}}} + \cancel{\Phi^X \Lambda^X \Phi^{X^{^{\prime}}}}+\frac{1}{J}\Phi^U \Lambda^U \Phi^{U^{^{\prime}}} +\\ &amp; \quad - 2\left(\cancel{\Phi^X \Lambda^X \Phi^{X}}+\frac{1}{J}\Phi^U \Lambda^U \Phi^{U^{^{\prime}}} \right)\\ &amp; = \Phi^U \Lambda^U \Phi^{U^{^{\prime}}} + \frac{1}{J}\Phi^U \Lambda^U \Phi^{U^{^{\prime}}} - \frac{2}{J}\Phi^U \Lambda^U \Phi^{U^{^{\prime}}}\\ &amp; = \left(1-\frac{1}{J}\right)\Phi^U \Lambda^U \Phi^{U^{^{\prime}}}\\ &amp; = \frac{J-1}{J} \Phi^U \Lambda^U \Phi^{U^{^{\prime}}}. \end{split} \end{gathered}\end{eqnarray*}

Thus, the separated estimator for the $U-$level score is

\begin{eqnarray*} \widehat\xi^U_{ij\ell} &amp; = &amp; \int_0^1 \widehat\lambda_\ell\widehat\phi_{\ell}^U\left(t\right)\left[\frac{\left(J-1\right)}{J}\left(\widehat R_U + \widehat\sigma^2_{\varepsilon_U} \textrm{I}_p\right)\right]^{-1}\widehat{Y_{ij}^U}\left(t\right) \mathrm{d}t.\end{eqnarray*}
